# Analyzing Thioflavin T Binding to Amyloid Fibrils by an Equilibrium Microdialysis-Based Technique

**DOI:** 10.1371/journal.pone.0030724

**Published:** 2012-02-24

**Authors:** Irina M. Kuznetsova, Anna I. Sulatskaya, Vladimir N. Uversky, Konstantin K. Turoverov

**Affiliations:** 1 Laboratory of Structural Dynamics, Stability and Folding of Proteins, The Institute of Cytology, Russian Academy of Sciences, St. Petersburg, Russia; 2 Department of Molecular Medicine, College of Medicine, University of South Florida, Tampa, Florida, United States of America; 3 Institute for Biological Instrumentation, Russian Academy of Sciences, Pushchino, Moscow Region, Russia; Consejo Superior de Investigaciones Cientificas, Spain

## Abstract

A new approach for the determination of the amyloid fibril – thioflavin T (ThT) binding parameters (the number of binding modes, stoichiometry, and binding constants of each mode) is proposed. This approach is based on the absorption spectroscopy determination of the concentration of free and bound to fibril dye in solutions, which are prepared by equilibrium microdialysis. Furthermore, the proposed approach allowed us, for the first time, to determine the absorption spectrum, molar extinction coefficient, and fluorescence quantum yield of the ThT bound to fibril by each binding modes. This approach is universal and can be used for determining the binding parameters of any dye interaction with a receptor, such as ANS binding to proteins in the molten globule state or to protein amorphous aggregates.

## Introduction

Benzothiazole dye thioflavin T (ThT) fluorescence is a well-known test for amyloid fibril formation in diseases associated with disturbances in protein folding, such as Alzheimer's and Parkinson's diseases, type II diabetes, and prion diseases. This approach is based on the unique property of ThT to form highly fluorescent complexes with amyloid and amyloid-like fibril [1], [2]. Except the acetylcholinesterase (AChE) [3] and serum albumins [4], ThT does not interact with globular proteins in a native state. It also does not interact with molten globule and unfolded states or amorphous aggregates of proteins. The study of the spectral properties of ThT [5] and the dependence of its fluorescence quantum yield on solution temperature and viscosity [6], [7], [8], together with quantum-chemical calculations of the dye molecule in the ground and excited states [9], have led us to believe that the significant increase in the fluorescence quantum yield when the dye is incorporated into the amyloid fibril is caused by a restriction of the torsion oscillations of ThT fragments relative to each other in the excited state [7], [8]. The Krebs model of ThT binding to amyloid fibril [10] suggests that the dye inserts itself into the grooves formed by side chains of amino acids composing β-sheets. It is now evident that the fibrils formed by different proteins, or even one protein in different conditions, are not identical [11], [12], [13], [14]. Apparently, the spectral characteristics of the ThT bound to fibril in different binding modes can also be different [15], [16], [17]. With this connection, the examination of the dye binding stoichiometry and affinity and characterization of its binding modes, i.e. the determination of binding parameters of ThT interaction with amyloid fibril, becomes an important problem. Since ThT can fluoresce only in the bound state (when incorporated into amyloid fibril), it seems natural to try to use ThT fluorescence to characterize its binding parameters. In almost all of the works focused on this problem, the binding constants were evaluated on the basis of the dependence of ThT fluorescence intensity on either ThT or fibril concentration. The results of these studies have been summarized in a review by Groenning [18].

The results from the aforementioned studies all assumed that the fluorescence intensity as a function of dye concentration reaches a plateau when all of the binding sites are occupied. Nonetheless, for any (even unbound) fluorophore, the fluorescence intensity dependence on the dye concentration is the curve with saturation. In this work, we demonstrate that a method based on dye fluorescence, in principle, could not provide reliable information on the concentration of the dye bound to fibril, and thus, this method cannot be used for determining the stoichiometry and affinity of the dye binding to fibril. To obtain information on the dye-receptor binding parameters we propose to use absorption spectrophotometry of solutions after equilibrium microdialysis. Surprisingly, this method, which is inherently designed for this purpose, has never been used for the determination of the binding parameters of ThT or its analogs to amyloid fibril.

This approach also allowed us to obtain information on the properties of ThT molecules incorporated into amyloid fibril, such as the absorption spectrum position, molar extinction coefficient, and quantum yield (if used together with registration of fluorescence intensity) of ThT bound to the amyloid fibril in different binding modes. The objects of investigation in this work were the insulin amyloid fibrils and AChE which is an exceptional protein which binds ThT in native state.

## Results

### Sample preparation for the absorption spectrophotometry determination of the equilibrium concentration of free and bound to fibril dye

Equilibrium microdialysis implies the allocation of two interacting agents, a ligand and receptor, in two chambers (#1 and #2, respectively) divided by a membrane permeable to the ligand and impermeable to the receptor. In our case, the ThT solution, with initial concentration *C_0_*, was placed in chamber #1 and the amyloid fibrils were placed in chamber #2. The content of the amyloid fibrils in chamber #2 was evaluated from the concentration of the protein solution used for amyloid fibril formation (*C_p_*). If the number of dye binding sites on the amyloid fibrils per protein molecule is *n*, then the concentration of binding sites is *nC_p_*. After reaching the equilibrium, the concentration of free ThT in chambers #1 and #2 becomes equal to *C_f_*, whereas the total ThT concentration in chamber #2 is greater than that in chamber #1 by the concentration of the bound dye (*C_b_*). Thus:
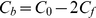
(1)


The initial concentration of ThT in chamber #1 (*C_0_*) and the dye concentration in this chamber after equilibration (*C_f_*), which equals to the concentration of the free dye in chamber #2, can be determined by absorption spectrophotometry. The concentrations of the dye bound to fibrils in chamber #2 can be determined on the basis of equation 1, and, consequently, the stoichiometry and affinity of ThT binding to amyloid fibrils can be evaluated. Furthermore, the use of equilibrium microdialysis also makes the measurement of spectral characteristics of the dye when incorporated into the amyloid fibril possible (see below).

### Absorption spectrum of ThT bound to amyloid fibril

**Figure 1:**
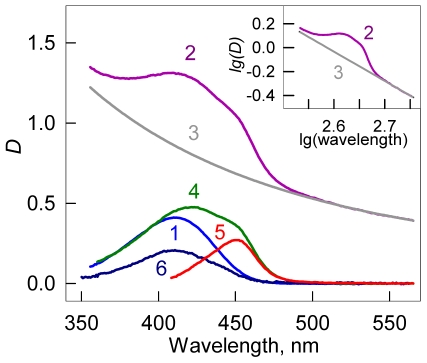
Absorption spectra of thioflavin T (ThT) incorporated in the amyloid fibril. Curves 1 and 2 represent absorption spectra of ThT in chamber #1 (free ThT at concentration *C_f_*) and in chamber #2 (superposition of the absorption spectra of free ThT in concentration *C_f_*, ThT bound to fibril in concentration *C_b_*, and the apparent absorption caused by the light scattering) after the equilibrium attainment. Curve 3 represents optical density determined by the fibril light scattering as calculated by the equation *D_scat_* = aλ^−m^. Coefficients a and m were determined from the linear part of the curve 2 (where there is no active dye absorption) plotted in logarithmic coordinates lg(*D_scat_*) = f(lg(λ)) (see **Insert**, curve 3). Curve 4 represents the total absorption of free and bound dye after light scattering subtraction (*D*(λ)_#2_−*D_scat_*). Curve 5 is the absorption spectra of ThT incorporated in the amyloid fibril evaluated as *D_b_*(λ) = *D*(λ)_#2_−*D*(λ)_scat_−*D*(λ)_#1_ (the difference between the spectra 4 and 1). Curve 6 is the absorption spectrum of the free dye at the concentration equal to that of bound dye (*D*(λ)_0_−2*D*(λ)_#1_). This curve allows for the evaluation of the change in the molar extinction coefficient of ThT when bound to fibril. Concentration of protein that was used for amyloid fibril preparation was *C_p_* = 6.9⋅10^−5^ M.

In equilibrium, the absorption spectrum of the solution in chamber #1 represents the absorption spectrum of free ThT of concentration *C_f_* (*D_f_* (λ)), while the absorption spectrum of the solution in chamber #2 represents the superposition of the absorption spectra of the free ThT (concentration *C_f_*), ThT bound to fibril of concentration *C_b_* (*D_b_*(λ)), and the apparent absorption determined by the light scattered by the fibril (*D_scat_*(λ)). The contribution of light scattering can be eliminated using the previously described methods [5]. Thus, in chambers #2 and #1, we have optimal sample and reference solutions for the accurate determination of absorption spectrum of ThT bound to fibril. The analysis of these spectra (see details in Figure 1) shows that the absorption spectra of ThT incorporated into insulin amyloid fibril (λ_max_ = 450 nm) are red-shifted in comparison to that of the free ThT (λ_max_ = 413 nm). It was also shown that the true absorption spectra of the bound ThT coincide with the excitation spectra of fluorescence recorded at 480 nm. The obtained data show that the molar extinction coefficients of ThT bound to different binding modes of insulin fibril are different, and that they differ from that of free ThT in solution.

### Binding parameters of ThT interaction with amyloid fibril

If all the ThT binding sites in amyloid fibril are identical and independent from each other, then the binding constant of the dye to amyloid fibril (*K_b_*) is determined as the ratio of the ligand-receptor complex concentration (*C_b_*) to the product of free receptor (*nC_p_−C_b_*) and free ligand (*C_f_*) concentrations:
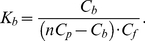
(2)


This means that the dependence of the bound dye concentration on the free dye concentration in solution follows a saturation curve:
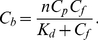
(3)


Here, 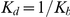 is the dissociation constant. Based on equations (1) and (2), the dependence of the concentration of bound dye (*C_b_*) on the initial dye concentration (*C_0_*) in chamber #1 can be calculated as:
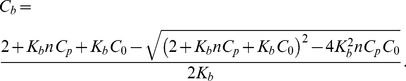
(4)


The value of the binding constant, *K_b_*, and the number of dye binding sites on the fibril in terms of protein concentration, *n*, can be determined on the basis of the experimental dependence of *C_b_* on *C_0_* (or *C_f_*) by non-linear regression using appropriate software, e.g. SigmaPlot or GraphPad Prism. The failure to find appropriate parameters means that the chosen model does not correspond to the experimental data. In particular, this mismatch can be attributed to the existence of two or more binding modes (*i*) with different binding constants (*K_bi_*). In this case, the binding sites are assumed to be independent from each other, 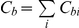, while *C_bi_* is characterized by the equations similar to those in (3). Therefore, in the case of *i* modes, we have:
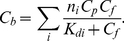
(5)The character of the Scatchard plot:
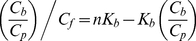
(6)or the Klots plot:
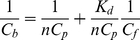
(7)can give the presentation of the number of binding modes.

**Figure 2:**
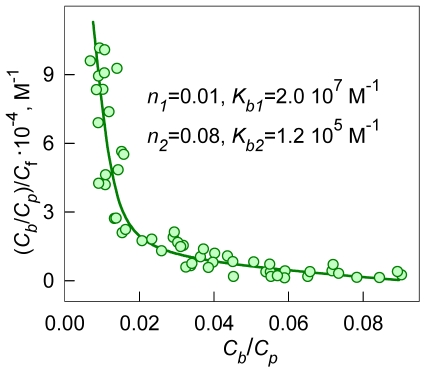
Scatchard plots for ThT interaction with insulin amyloid fibril. Experimental data (circles) and best fit curve with binding constants (*K_bi_*) and number of binding sites (*n_i_*) given on the panels.

The nonlinear dependence of *C_b_* on *C_f_* in Scatchard coordinates obtained for ThT binding to insulin fibril (Figure 2), and the failure of equations (3) and (4) to describe the experimental data, suggests that the amyloid fibrils formed by insulin have more than one binding mode with significantly different binding constants. To adequately describe the experimental data, a value of *i* = 2 was assumed and the values of *K_bi_* and *n_i_* were found by fitting the data to eq. 5 using GraphPad Prism. These values are given in Figure 2 and in Table 1. For AChE, which has one ThT binding center, just one ThT binding mode was found (Table 1).

**Table 1:** Characteristics of thioflavin T bound to amyloid fibrils, acetylcholinesterase and free dye in water solution.

Object	*λ_max_*, nm	*mode*	*ε_i_*, _max_×10^−4^, M^−1^ cm^−1^	*ε_i_*, _435_×10^−4^, M^−1^ cm^−1^	*K_bi_*×10^−5^, M^−1^	*n_i_*	*q_i_*
Insulin fibrils	450	1	8.7	5.7	200	0.01	0.83
		2	3.5	2.6	1.2	0.08	0.30
Lysozyme fibrils1)	449	1	5.1	3.7	75	0.11	0.442)
		2	6.7	5.8	0.56	0.24	5×10^−42)^
Acetylcholinesterase	420	1	2.4	1.9	0.082	1.1	0.036
Thioflavin T in water solution3)	412	-	3.2	2.0	-	-	0.0001

1) Sulatskaya *et al*. 2011 [31].

2) unpublished data.

3) Sulatstaya *et al*. 2010 [8].

### Molar extinction coefficient of ThT bound to amyloid fibril

In the case of one binding mode, the measured absorption spectrum can be easily presented in the units of the molar extinction coefficient:
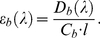
(8)


If there are two binding modes, then the concentrations of dye found to each mode can be calculated on the basis of the *K_d1_*, *K_d2_*, *n*_1_, and *n*_2_ values:
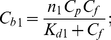
(9)

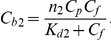



**Figure 3:**
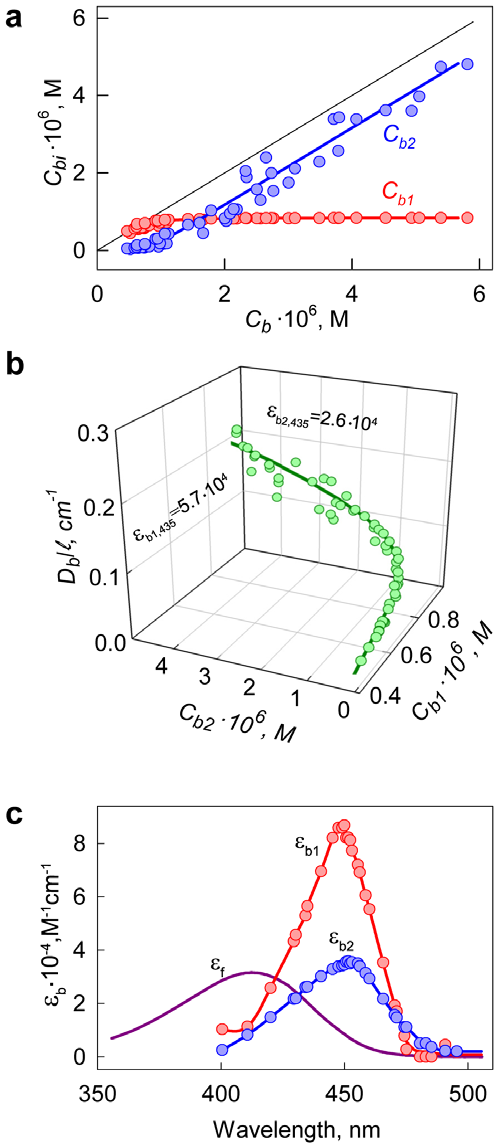
Determination of the molar extinction coefficient of thioflavine T (ThT) bound to insulin amyloid fibril. (a) Concentration of ThT, bound with amyloid fibril (*C_b_*), as superposition of the dye concentrations bound to mode 1 (*C_b1_*) and mode 2 (*C_b2_*). (b) The dependence *D_b_* = *D_b1_*+*D_b2_* = ε_b1_ *C_b1_ l*+ε_b2_ *C_b2_ l*. In the panel experimental data, best fit curve and the values of molar extinction coefficients ε_b1_ and ε_b2_ obtained by multiple nonlinear regression are presented. (c) Absorption spectra of ThT, bound to mode 1 and mode 2 in the units of the molar extinction coefficient.

Figure 3a shows the decomposition of *C_b_* into two components, *C_b1_* and *C_b2_*. Taking into account that
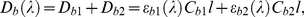
(10)the values of *ε_b1_(λ)* and *ε_b2_(λ)* can be determined using the known values of *D_b_(λ)*, *C_b1_*, and *C_b2_* by multiple linear regression (e.g. using SigmaPlot). Figure 3b shows the relation between *D_b_* at 435 nm and *C_b1_* and *C_b2_* values that result in values of *ε_b1_*(435) and *ε_b2_*(435) for insulin amyloid fibril. Similarly, the values of *ε_b1_* and *ε_b2_* can be determined at the other wavelengths. Figure 3c shows the absorption spectra of the dye bound to each of the two modes of insulin amyloid fibril in units of the molar extinction coefficient. This data showed that the molar extinction coefficient of ThT bound to fibril depends on the binding mode and can be significantly greater than that of the free dye in solution (Table 1).

### The determination of the quantum yield of ThT bound to fibril

One of the important characteristics of ThT bound to the amyloid fibrils is its fluorescence quantum yield. Although it is known that the fluorescence intensities of ThT bound to amyloid fibrils formed by different amyloidogenic proteins can be noticeably different, there are no literature data on the fluorescent quantum yield of ThT bound to amyloid fibrils. In this section show how the fluorescent quantum yield of ThT bound to amyloid fibrils can be determined on the basis of the fluorescence intensity of solutions prepared by equilibrium microdialysis and analogous registration of fluorescence intensity of the solution of the dye with the known quantum yield. In this work, the fluorescent dye ATTO-425 with the known quantum yield was used as an etalon.

The solution of ThT in the presence of amyloid fibril is a two-component system in which one component (free ThT unbound to fibril) absorbs the excitation light (optical density, *D_f_*) but does not fluoresce, while the other component (ThT bound to fibril) absorbs the excitation light (optical density, *D_b_*) and fluoresces (quantum yield, *q_b_*). Therefore:
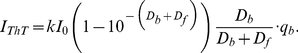
(11)


Here, *I_0_* is the intensity of the excitation light and *k* is the proportionality coefficient. Fluorescence intensity can be normalized (*kI_0_* = 1) so that *I_ThT_ = q_b_ D_b_*/(*D_b_+D_f_*) at (*D_b_+D_f_*)→∞ and, consequently, *I_ThT_ = q_b_* at *D_b_*→∞ and *I_ThT_ = 0* at *D_f_*→∞. In equation (11), two factors can be selected. One factor is the function of only the total optical density of the solution, and does not depend on the contribution of the optical density of the fluorescent component:
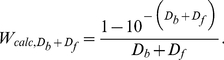
(12)


The other factor is a product of optical density and the quantum yield of the fluorescent component (*D_b_q_b_*). Then equation (11) can be written as follows:
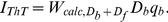
(13)


The fluorescence intensity of a single-component solution can also be presented in the same manner. In particular, for a solution of ATTO-425, which in this work is used as a standard substance with known quantum yield, the fluorescence intensity can be presented as follows:
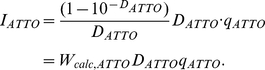
(14)


**Figure 4:**
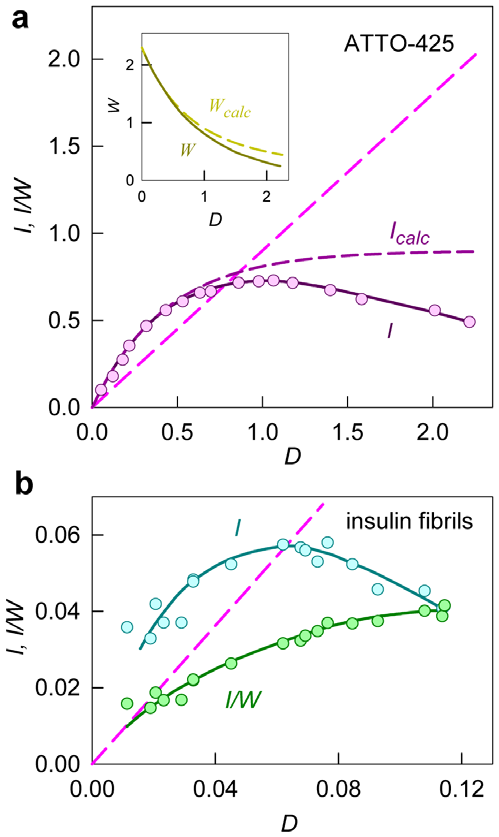
The dependence of fluorescence intensity on optical density of fluorophore and on total optical density of solution. (**a**) The dependences of fluorescence intensity on optical density (*D_ATTO_*) of the fluorescence dye ATTO-425 with known quantum yield (*q_ATTO_* = 0.9) calculated from equation (15) and experimentally recorded *I*. The dependence of fluorescence intensity on the optical density of the fluorescent substance (*I*) differs from the calculated one (*I_calc_*) because an increase in the total optical density of the solution results in the increased absorption of the excitation light by the solution layers adjacent to the front wall of the spectrofluorometer cell, while the detecting system of spectrofluorometer “see” the central part of the cell, which is reached by a respectively smaller part of the excitation light. Due to this effect, the recorded fluorescence intensity begins to diminish after optical density reaches some value. The discussed effects depend on the instrument used and must also be taken into account. This can be accomplished by replacing *W_calc_* with *W*, which depends on total optical density of the solution and can be determined experimentally. As for *W_calc_*, the value of *W* is determined only by the total optical density of solution and does not depend on the contribution of the optical density of the fluorescent substance. The dependences of *W_calc_* and *W* on total optical density are given in the **Insert**. The strait dashed line is the dependence of *D_ATTO_q* on *D_ATTO_* calculated as *D_ATTO_q = I_calc_/W_calc_ = I/W*. (**b**) The dependencies of the experimentally recorded fluorescence intensity (*I*) and the reduced fluorescence intensity (*I/W*) of ThT bound to insulin fibril on its optical density (*D_b_*). The strait dashed line is the dependence of *D_ATTO_q* on *D_ATTO_* for the fluorescence dye ATTO-425 (etalon with *q_ATTO_* = 0.9).

It is evident that the dependence *I_ATTO_ = f(D_ATTO_)* is a curve with saturation (*I_ATTO_* = *q_ATTO_* at *D_ATTO_*→∞), while the dependence *I_ATTO_/W_calc,ATTO_ = f* (*D_ATTO_*) is a straight line with a slope equal to the fluorescent quantum yield of the dye (Figure 4).

The experimental dependence of fluorescence intensity on the optical density of the fluorescent substance can differ from the calculated one (Figure 4). Hence, in equations (13) and (14) the value of *W_calc_* must be replaced with *W*, which, similar to *W_calc_*, depends only on the total optical density. Using equations (13) and (14), in which *W_calc_* is replaced by *W*, one can write:
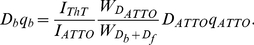
(15)


To exclude the influence of the factor depending on the total optical density it is necessary to record the fluorescence intensity of the fluorescent dye ATTO-425 solution (etalon with known quantum yield) whose optical density equals to the summarized optical densities of free and bound to fibril ThT. In this case:
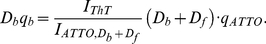
(16)


The value of *D_b_q_b_* is determined as an average of several values independently measured for samples with different dye concentrations after microdialysis. In the case of two binding modes, a relation similar to equation (16) is valid:
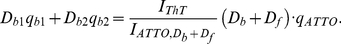
(17)


To determine the values of *q_b1_* and *q_b2_*, it is necessary to know the set of three related values *D_b1_*, *D_b2_*, and (*D_b1_q_b1_*+*D_b2_q_b2_*) that correspond to one microdialysis experiment and that are related to each other by equation (17).

**Figure 5:**
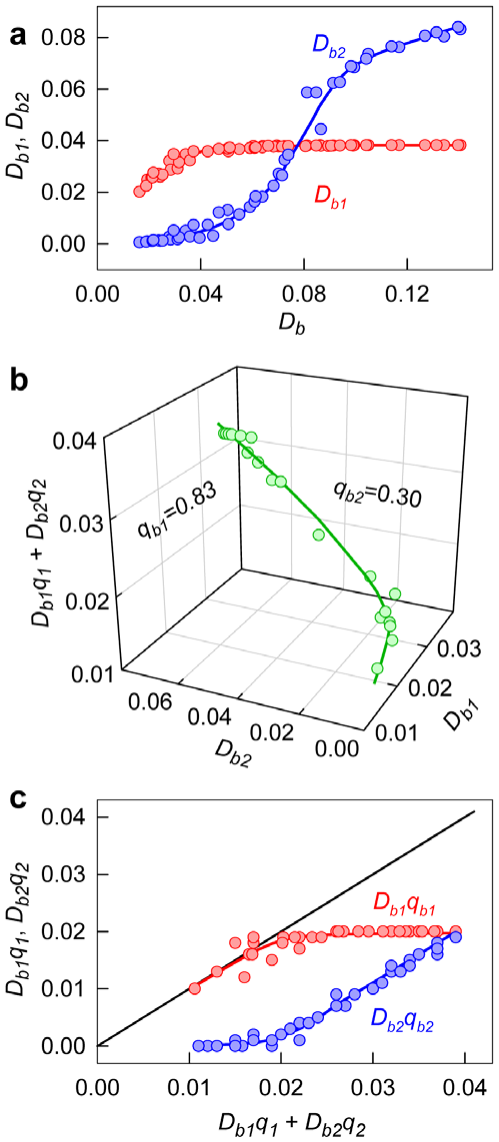
Determination of the fluorescence quantum yield of thioflavine T (ThT) bound to fibril. (a) Optical density of ThT bound to amyloid fibril as superposition of optical densities of the dye bound in mode 1 (*D_b1_*) and mode 2 (*D_b2_*). (b) 3D dependence of *D_b1_q_b1_*+*D_b2_q_b2_* from *D_b1_* and *D_b2_*. Experimental data, best fit curve and the values of *q_b1_* and *q_b2_*, determined multiple nonlinear regression are presented. (c) Reduced fluorescence intensities of ThT/W as superposition of the reduced fluorescence intensities of the dye bound to mode 1 (*D_b1_q_b1_*) and mode 2 (*D_b2_q_b2_*).

The dependences of the values of *D_b1_ = ε_b1_C_b1_ l* and *D_b2_ = ε_b2_C_b2_ l* on *D_b_* can be determined on the basis of the values of the molar extinction coefficients and concentrations of ThT bound to two modes, and are determined for the samples obtained after microdialysis (Figure 5a). The dependence of the value (*D_b1_q_b1_*+*D_b2_q_b2_*) on *D_b_* can be determined from equation (17). On the basis of these data, the fluorescence quantum yields of the dye bound to each mode can be determined using multiple nonlinear regression (Figure 5b, Table 1). The contribution of the fluorescence intensity of ThT bound to each mode in the total fluorescence intensity is presented in Figure 5c.

## Discussion

Recently, the utility of studying the amyloid fibril structure by investigation of their interaction with ThT became evident [18]. Therefore, the accurate determination of the binding parameters of the ThT (or its derivatives and analogs) interaction with the amyloid fibril is a very important task. Practically all existing data about it are based solely on the measurements of the ThT fluorescence intensity dependence on the dye concentration in solutions containing amyloid fibril. The data represented in this work show the failure of such an approach. Experiments based on fluorescence intensity measurement, in principle, cannot provide information about the concentration of free dye. Nonetheless, researchers used equations (3) or (7), replacing the value of free dye concentration with the value of introduced (total) dye concentration, for the determination of the binding constants, without any explanation of such a change (see [1], [19], [20], [21], [22]). This problem was mentioned in the Supplement to the work by Sutharsan *et al*. [22], but was left otherwise unaddressed. We believe that the replacement of the concentration of free dye with the concentration of total dye in equations (3) and (7) contradict to the physical meaning of the current task.

Earlier [3], the dependence of fluorescence intensity of ThT on the *C_0_* was analyzed using an equation similar to equation (4), which is more correct. However, there is still a problem of how fluorescence intensity depends on *C_b_*. The determination of the stoichiometry and affinity of ThT binding to amyloid fibril by fluorescence was based on the assumptions that the recorded fluorescence intensity is always proportional to the concentration of bound dye (*I* = *kC_b_*), and that fluorescence intensity plateaus when all binding sites are occupied (*I*_max_ = *knC_p_*) [19]. We showed that both of these assumptions are inaccurate (see equations (11) and (14)). Even experienced researchers, who do not specialize in fluorescence techniques, do not take into account the fact that a plateau of the dependence of fluorescence intensity on the optical density of a fluorescent substance could not point to the saturation of binding centers since, such a character of the dependence is its general property.

Furthermore, the recorded fluorescence intensity could even decrease with an increase in the content of the fluorescent substance which is also a general property of such dependences (Figure 4) and does not necessitate the existence of self-quenching as it was suggested in the work [23]. In reality, the fluorescence intensity is determined not only by the concentration of the bound dye but also by the total optical density of the solution. The fluorescence intensity corrected for the factor *W* depending on the total optical density of solution is the product of the optical density and fluorescence quantum yield of the dye bound to fibril: *I/W = q_b_D_b_ = q_b_ε_b_C_b_*. Therefore, even in the case of one binding mode, a special assumption must be made for the values of *q_b_* and *ε_b_*, which, as we showed, can differ from these values of free dye. In the case of two binding modes, the use of fluorescence intensity for the determination of binding parameters is even more difficult because in this case, 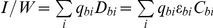.

We have shown that absorption spectrophotometry determination of the concentration of free and bound to fibril dye in solutions, which are prepared by equilibrium microdialysis, can give direct and accurate data on the number of binding modes and stoichiometry and affinity of each mode of ThT binding to amyloid fibril. Measurement of the fluorescence intensity of the same solutions permit the determination of the fluorescence quantum yield of ThT bound to amyloid fibril in a definite binding mode. The main results concerning insulin amyloid fibril obtained in this work from examination their interaction with ThT are follows:

1. For the first time, the absorption spectrum of ThT bound to amyloid fibril was recorded. It was found to be significantly red shifted (λ_max_ = 449–450 nm) in comparison to free ThT in solution (λ_max_ = 412–413 nm). As expected, this spectrum coincided with the fluorescence excitation spectrum of bound dye. The significantly shorter wavelength of the absorption spectrum of free ThT in solution, in comparison with that of ThT incorporated into amyloid fibril, can be explained by the orientational dipole–dipole interaction of the dye molecules with a polar solvent (see [5], [7]).
2. The stoichiometry and affinity of ThT binding to different binding modes differs significantly. The existence of several dye binding modes in amyloid fibril was shown in many works (see, the review by Groenning [18]). The possibility of ThT interactions with Aβ amyloid fibril in several binding modes was shown by molecule dynamic simulations [16], [17].
3. ThT binding to fibril is accompanied not only by a significant red shift of the absorption spectrum, but also by a significant change in the molar extinction coefficient. This result strongly agrees with the quantum chemical simulations that predict the dependence of the value of the oscillator strength corresponding to the ThT transition from the ground to the excited state on the *ϕ* value between the benzothiazole and aminobenzoyl rings [9] and with the assumption that conformation of ThT molecules bound to amyloid fibril (value of *ϕ*) may differ from that of free molecule in solution [8].
4. For the first time, the molar extinction coefficients of ThT bound to insulin amyloid fibril in each binding mode were evaluated.
5. Using the value of optical density and the fluorescence data, the fluorescence quantum yield of the ThT bound to fibril in each binding mode was determined (Table 1). As it was predicted in the work of Sulatskaya *et al*. 2010 [8], the fluorescence quantum yield of ThT incorporated into amyloid fibrils depends not only on the restriction of high frequency rotational oscillations of ThT molecule fragments against each other in the excited state, but also on the molecular conformation of ThT in the ground state, and could be both larger or smaller than quantum yield in rigid isotropic solution (*q* = 0.28). It was shown that in the case of insulin fibril, the ThT molecules bound with higher binding constants had a noticeably higher fluorescence quantum yield (*q*_b1_ = 0.83). At the same time, the fluorescent quantum yields of ThT bound to lysozyme in the second binding mode (which is characterized by the low binding constant) was comparable to that of free ThT in aqueous solution. The fluorescent quantum yield of ThT bound to AChE was higher than that of ThT in aqueous solution but still was an order of magnitude lower than the fluorescent quantum yield of ThT in the rigid isotropic solution (Table 1).
6. The proposed approach was also applied to AChE that is an exceptional protein active center of which is known to bind ThT in its native state [3]. As expected, just one binding constant was determined (Table 1), which, as expected, was much smaller than the ThT binding constant to amyloid fibrils.

In brief, the obtained results show that the use of equilibrium microdialysis opens new possibilities for the ThT application in investigation of amyloid fibril structure. The proposed approach is universal for determining the binding paramenters of any dye to a receptor. An accurate determination of the binding parameters of neutral ThT analogs, which can penetrate the hematoencephalic barrier, to amyloid fibril might have considerable consequences for the successful development of the diagnosis and therapy of neurodegenerative diseases [21], [22], [24], [25], [26], [27], [28]. Surprisingly, the approach proposed in this work, which is inherently designed for the binding parameter determination for the ligands binding to receptors, was never used in studying the interaction of ThT or its analogs to amyloid fibril.

## Materials and Methods

Thioflavin T (ThT) from Sigma (USA) and Fluka (Switzerland) were used after purification by crystallization from a mixture of acetonitrile with ethanol in the ratio 3∶1 [6]. ThT “UltraPure Grade” from AnaSpec (USA) were used without afterpurification. ThT was dissolved in 2 MM Tris-HCl buffer (pH 7.7), with 150 MM NaCl. The samples of insulin and buffer components from Sigma (USA) were used without afterpurification. Insulin amyloid fibrils were prepared as described earlier [29]. Acetilcholineaterase *Electrophorus electricus* (electric eel) from Sigma (USA) was dissolved in 20 mM sodium phosphate buffer (pH 7.0), protein concentration was 1.53 mg/ml.

The absorption spectra were recorded by spectrophotometer U-3900H (Hitachi, Japan). Fluorescence measurements were performed using the homemade spectrofluorimeter [30] and spectrofluorimeter Cary Eclipse (Varian, Australia). Equilibrium microdialysis was done with the Harvard Apparatus/Amika (USA) device. It consists of two chambers (500 µL each) separated by a membrane (MWCO 10 000) impermeable for particles larger than 10 000 Da. For performing equilibrium microdialysis the devices were set on rocking-bar in the thermostated box for 24 hours. Afterwards, the absorption spectra of solutions from chamber # 1 and # 2 were determined.

To prove that the microdialysis for 24 h is sufficient for the ThT equilibration between chambers #1 and #2, and to prove that ThT does not interact with membrane or chambers walls, the following test experiments were performed. We put solution of ThT in concentration *C*_0_ in chamber # 2 and the dye-free solvent in chamber # 1 and followed establishing of the equilibrium by the periodical measurements of the absorption spectra in both chambers. These experiments revealed that after the dialysis for just 10 hours, the absorption spectra of solutions from # 1 and # 2 coincided (*D*(λ)_#1_ = *D*(λ)_#2_). This meant that the utilized in this work dialysis for 24 h was sufficient for the dye equilibrium distribution between the chambers # 1 and # 2. In addition it was shown that *D*(λ)_#1_ = *D*(λ)_#2_ = *D*(λ)_0_/2, clearly illustrating that the dye did not bind to membrane and chambers' walls. In additional control experiments we established that the decrease in the microdialysis duration to 10 h or its increase to 48 h did not change the concentration of free dye in chamber #1, suggesting complete equilibration of free and bound dye in chamber #2. All experiments were done at 23°C. Absorption spectra were recorded by spectrophotometer U-3900H (Hitachi, Japan). Optical densities of free and bound dye were determined as described earlier [31].
